# Curcumin hormesis mediates a cross-talk between autophagy and cell death

**DOI:** 10.1038/cddis.2015.343

**Published:** 2015-12-03

**Authors:** N Rainey, L Motte, B B Aggarwal, P X Petit

**Affiliations:** 1 INSERM U1124, Toxicology, Experimental pharmacology and Signal transduction, Université Paris-Descartes, 45 Rue des Saints-Pères, Paris 75270, France; 2 Université Paris 13, Sorbonne Paris Cité, UFR de Santé, Médecine et Biologie Humaine, Bobigny 93017, France; 3 Cytokine ResearchLaboratory, Department of Experimental Therapeutics, The University of Texas MD Anderson Cancer Center, 1901 East Road, Unit 1950, Houston, TX 77054, USA

Curcumin is one of the most successful compounds investigated in recent years, and is currently being assessed both for prevention and treatment of cancer.^1^ Curcumin can exist in at least two tautomeric forms, keto and enol. The enol form is more energetically stable in the solid phase as well as in solution. Curcumin incorporates several functional groups. The aromatic ring systems, which are polyphenols, are connected by two *α*-*β*-unsaturated carbonyl groups. The diketones form stable enols or, because they are easily deprotonated, form enolates, whereas the *α*-*β*-unsaturated carbonyl groups are a good Michael acceptor and undergoes nucleophilic addition. Most of the know activities of curcumin have been assigned to its methoxy, hydroxyl, *α*-*β*-unsaturated carbonyl moiety or diketone groups. Curcumin may disrupt disulfide bond formation by the electrophilic dienone. Free thiols on cysteine-rich proteins are available to react with Michael acceptors of curcumin.^2^

Frequently, low doses of toxins and other stressors not only are harmless but also activate an adaptive stress response that raise the resistance of the organism against high doses of the same agent.^3^ This phenomenon is referred to as hormesis. Many molecules that cause cell death also elicit autophagy, a cytoprotective mechanism relying on the digestion of potentially harmful intracellular structures, notably mitochondria. When higher doses of these agents are employed, cells undergo mitochondrial outer membrane permeabilization and die.

Driven by the recent literature on hormetic phytochemicals, we focused our attention on curcumin, which is known to mediate beneficial effects on health even for a better understanding of its cellular internalization, intracellular localization and mode of action. A plethora of publications proved that curcumin can bind and/or inhibit numerous targets including, for example, Nrf2, *β*-catenin, NF-*κ*B, inducible nitric oxide synthase, nitric oxide, amyloid plaques, reactive oxygen species (ROS), cyclin D1, glutathione, cytosolic phospholipase A2, inhibitor of NF-*κ*B kinase-1-2, P38MAPK, p-Tau (p-*τ*) and tumor necrosis factor-*α*. Curcumin inhibit tumor cell growth and suppress cellular entry of viruses very potently,^4^ and is efficient in suppressing phorbol-ester-induced tumor promotion.^5^ Curcumin insertion into intracellular membranes affects membrane mobility and permeability, also acting on ion channels and transporters. But the main characteristics of curcumin, justifying its hormesis capability, is to possess both antioxidant and pro-oxidant properties that are closely related to autophagic and cell death activation processes.

In a recent article published in *Cell Death Discovery*, Moustapha *et al.*^6^ have depicted the molecular circuitries that link curcumin to cellular stress and death, and how these pathways can get uncoupled during hormetic responses. We report that curcumin at very low doses (≤1 *μ*M) is indeed an excellent antioxidant but that medium doses of curcumin (in the range of 5–10 *μ*M) operates primarily as an autophagy inducer, correlating with their described capacity to reduce the acetylation of cytoplasmic proteins^7^ and cell cycle blocker. Finally, at further higher doses (over 25 *μ*M), cell death is induced (all experiments run for 48 h). We investigated mechanistic aspects of the destabilization of the endoplasmic reticulum (ER) and lysosome involved in mitochondrially associated apoptosis. Curcumin induces an ER stress causing calcium release which in turn destabilizes the mitochondrial compartment to induce apoptosis. These events are also associated with lysosomal membrane permeabilization and activation of caspase-8, mediated by activation of cathepsins and calpains. This complex interplay is of huge interest, as the efficient autophagy may allow cells to escape the G2/M blockage^6, 8^ induced by curcumin when used at 10 *μ*M.

It has been suggested that it may be possible to exploit autophagy for cancer therapy. After treatment of Huh7 cells with curcumin, a complex interplay involving early autophagy is induced as soon as mitochondria produce superoxide anions and hydrogen peroxide. Induction of autophagy, marked by the formation of autophagic vacuoles,^6^ was followed by staining with acridine orange and monodansylcadaverine dye after exposure to 10 *μ*M curcumin. At this concentration only early events of apoptosis are detectable. Western blotting following curcumin treatment showed the conversion of LC3-I to LC3-II, a marker of active autophagosome formation. We also found that the production of ROS and formation of autophagic vacuoles following curcumin treatment was almost completely blocked by each of *N*-acetylcystein, the mitochondrial-targeted antioxidants, MitoQ or SKQ1, the calcium chelator, BAPTA-AM, and the mitochondrial calcium uniport inhibitor, ruthenium red (Figure 1).

Our recent findings describe the mechanism through which curcumin promotes selective tumor cell death, providing unequivocal evidence of the role of the ER and lysosomal destabilization together with an intricated cross-talk between autophagy and cell death in contrasting the proliferation of a human hepatocellular carcinoma cell line (Huh7 cells). These results fit well with the fact that other mechanistic investigations revealed that curcumin treatment also upregulated the ER stress markers CHOP and Bip/GRP78 (ref. 9) and, as we described, the autophagic marker LC3-II. In addition, curcumin induced ER stress by triggering ROS generation, which was supported by the finding that treating cells with the antioxidant *N*-acetylcystein alleviated curcumin-mediated ER stress and vacuolation-mediated death.

Autophagy, a lysosomal degradation pathway for cellular constituents and organelles, is an adaptive and essential process required for cellular homeostasis. Although autophagy can also lead to a non-apoptotic form of programmed cell death called autophagy-induced cell death or autophagy-associated cell death. Thus, with these results,^6^ we are entering straight in the main ongoing discussions about the curcumin capability to induce autophagic processes and to evaluate its potency. Either being a complete autophagy with autophagolysosome formation or an intense vacuolization with no fusion with the lysomomes and ceramide accumulation.^10^

All these recent findings suggest that curcumin or curcumin derivatives have the potential to be developed into pro-autophagic drugs for the treatment of cancers^1, 11^ and malignant gliomas.^10^ Various formulations of curcumin, including nanoparticles, liposomal encapsulation, emulsions, capsules, tablets and powder, have been examined. The capacity of these compounds to provide a means of cancer cell death that enhances the effects of standard therapies should be taken into consideration for designing novel therapeutic strategies. Moreover, and alternatively, the regulation of autophagy by polyphenolic coumpounds^12^ if controlled should be considered as a potential therapeutic strategy for cancer.^1^

## Figures and Tables

**Figure 1:**
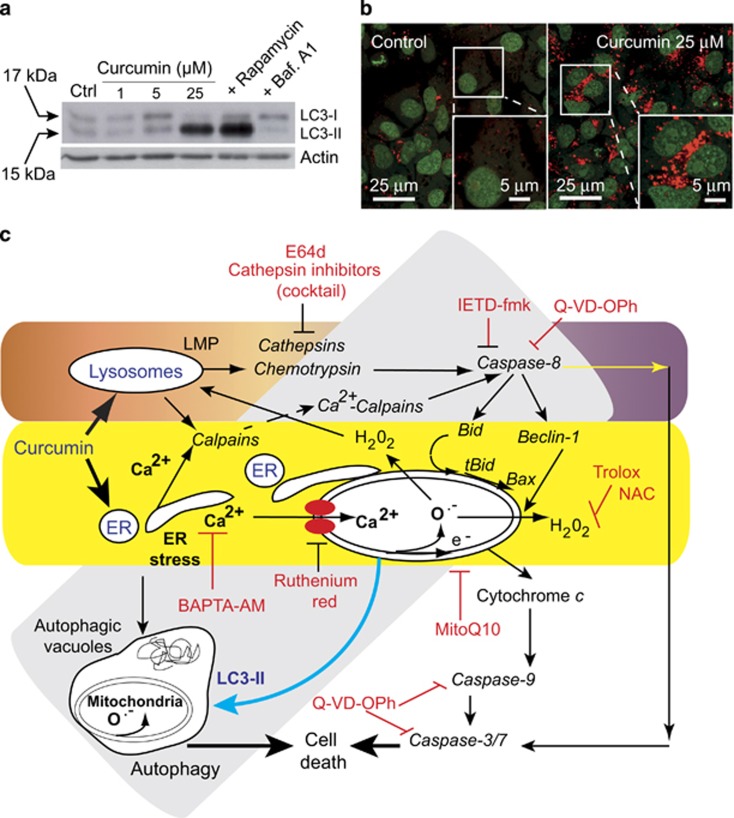
(**a**) Western blot analysis of the conversion of LC3-I to LC3-II in cells treated for 24 h with different curcumin concentrations (1, 5 or 25 *μ*M). Control are: treatment of the cells with rapamycin 1 *μ*M to induce autophagy and treatment with bafilomycin A_1_ 0.5 *μ*M to inhibit it. (**b**) Confocal microscopy of acridine orange (AO)-stained vesicles in cells treated with curcumin at 25 *μ*M compared with the control. In the section showing cells treated with 25 *μ*M curcumin, the enlarged panel (lower right panel) reveals the massive accumulation of AO-positive big vesicles with an acidic content that are remisniscent of autophagosomes. (**c**) Curcumin mainly targets the endoplasmic reticumum (ER) and lysosomes. The classic apoptotic pathway is mediated by calcium release from the ER. Uptake of this calcium by mitochondria disrupts mitochondrial homeostasis. Calcium alters mitochondrial electron transport causing substantial ROS production (both superoxide anions and hydrogen peroxide), which leads to the opening of the permeability transition pore in the mitochondrial membrane. Consequently, cytochrome *c* is released and the caspase-9 and caspase-3/7 pathway is activated leading to cell death. Furthermore, the ER stress pathway leads to the formation of autophagic vacuoles that attempt to eliminate the dysfunctional mitochondria. The cleavage of Beclin-1 is associated with early apoptosis and leads to the accumulation of autophagic vacuoles. So, despite the activation of autophagy, cells undergo a type of ‘necrotic cell death' following these initial apoptotic events. These two pathways are parralled by a lysosomal pathway. Indeed, curcumin destabilizes lysosomal membranes leading to lysosomal membrane permeability and the activation of both cathepsins and chemotrypsins. Activated caspase-8 leads to Beclin-1 cleavage that inhibits the primarily induced autophagy. The increase in cytosolic calcium concentration also activates calpains that contribute to the degradation process and accelerate cell death. The various inhibitors used in this work are indicated at the place where the pathways are affected
